# Transiently impaired endothelial function during thyroid hormone withdrawal in differentiated thyroid cancer patients

**DOI:** 10.3389/fendo.2023.1164789

**Published:** 2023-06-22

**Authors:** Li-ying Hou, Xiao Li, Guo-qiang Zhang, Chuang Xi, Chen-tian Shen, Hong-jun Song, Wen-kun Bai, Zhong-ling Qiu, Quan-yong Luo

**Affiliations:** ^1^ Department of Nuclear Medicine, Shanghai Sixth People’s Hospital Affiliated to Shanghai Jiao Tong University School of Medicine, Shanghai, China; ^2^ Department of Ultrasound in Medicine, The Sixth People's Hospital Affiliated to Shanghai Jiao Tong University School of Medicine, Shanghai, China; ^3^ Shanghai Institute of Ultrasound in Medicine, Shanghai, China

**Keywords:** endothelial dysfunction, lipids, differentiated thyroid cancer, radioiodine therapy, flow-mediated dilation

## Abstract

**Purpose:**

Endothelial dysfunction, which was associated with chronic hypothyroidism, was an early event in atherosclerosis. Whether short-term hypothyroidism following thyroxine withdrawal during radioiodine (RAI) therapy was associated with endothelial dysfunction in patients with differentiated thyroid cancer (DTC) was unclear. Aim of the study was to assess whether short-term hypothyroidism could impair endothelial function and the accompanied metabolic changes in the whole process of RAI therapy.

**Methods:**

We recruited fifty-one patients who underwent total thyroidectomy surgery and would accept RAI therapy for DTC. We analyzed thyroid function, endothelial function and serum lipids levels of the patients at three time points: the day before thyroxine withdrawal(P_1_), the day before ^131^I administration(P_2_) and 4-6 weeks after RAI therapy(P_3_). A high-resolution ultrasound named flow-mediated dilation (FMD) was used to measure endothelial function of the patients.

**Results:**

We analyzed the changes of FMD, thyroid function and lipids at three time points. FMD(P_2_) decreased significantly compared to FMD(P_1_) (P_1_vsP_2_, 8.05 ± 1.55vs 7.26 ± 1.50, p<0.001). There was no significant difference between FMD(P_3_) and FMD(P_1_) after restoring TSH (thyroid stimulating hormone) suppression therapy (P_1_ vs P3, 8.05 ± 1.55 vs 7.79 ± 1.38, p=0.146). Among all parameters, the change of low-density lipoprotein (ΔLDL) was the only factor correlated negatively with the change of FMD (ΔFMD) throughout the RAI therapy process (P_1-2_, r=-0.326, p=0.020; P_2-3_, r=-0.306, p=0.029).

**Conclusion:**

Endothelial function was transiently impaired in DTC patients at short-term hypothyroidism state during the RAI therapy, and immediately returned to the initial state after restoring TSH suppression therapy.

## Introduction

The frequency of diagnosis of differentiated thyroid cancer (DTC), the most common subtype of thyroid cancer and the most common endocrine malignancy, continues to rise as a result of the widespread use of diagnostic imaging and surveillance (1–3). However, the mortality rate associated with DTC remains low, relative to that associated with other cancers, because of the indolence of thyroid cancer and the availability of effective treatments (4). Radioiodine (RAI) treatment is an effective method of treating potential remnant cancer tissue and metastases following surgery (5). High concentrations of thyroid stimulating hormone (TSH) (>30 mIU/L) are required for RAI therapy to be performed, because this increases the ^131^I uptake by thyroid cancer tissue. A sufficient serum TSH concentration is usually achieved in clinical practice by the withdrawal of thyroid hormone for 3–4 weeks. Therefore, patients experience short-term hypothyroidism during RAI therapy (6).

The association between hypothyroidism and cardiovascular (CV) diseases is well established, with reports dating as far back as 1878, when thyroid function tests were not available (7). It has been shown that patients with overt hypothyroidism frequently experience pericardium effusion, cardiac enlargement, and atherosclerosis (8). Hypothyroidism has been demonstrated to be an independent risk factor for atherosclerosis (9), the potential mediators of which include dyslipidemia, hypercoagulability, arterial stiffness, obesity, and endothelial dysfunction (10). The endothelial dysfunction, first reported in 1995, is an early stage in the development of atherosclerosis, and is associated with a higher risk of CV events (11). The endothelium plays important roles in modulating vascular tone by synthesizing and releasing an array of endothelium-derived relaxing factors, including vasodilator prostaglandins, NO, and endothelium-dependent hyperpolarization factors, as well as endothelium-derived contracting factors (12). Endothelial cells, forming a continuous monolayer along the inner surface of arteries, are activated by thyroid hormone through its binding to thyroid hormone receptors, which are expressed on both the myocardial and vascular endothelium. This leads to the production of nitric oxide (NO), the most important vasodilator substance, causing the relaxation of vascular smooth muscle cells and arterial dilatation (12–15). The relationship between hypothyroidism and endothelial dysfunction has now been thoroughly studied. It was well established that chronic hypothyroidism caused endothelial dysfunction by reducing NO availability (16–18). And subclinical hypothyroidism patients, patients with a TSH level>10 mIU/L and normal thyroid hormone levels, had a higher risk of developing heart failure (19). But it is unclear whether short-term hypothyroidism has similar effects like subclinical hypothyroidism or chronic hypothyroidism patients.

Endothelial function can be assessed using flow-mediated dilation (FMD) that measures flow-mediated changes in brachial artery diameter induced by short term ischemia. Flow-mediated changes are endothelium-dependent, largely NO-mediated dilatation of arteries in response to artificially induced increases in blood flow induced shear stress. The brachial artery is occluded by the inflated cuff for 5 min, and the shear-induced NO would increase and the vasodilation of the brachial artery would be induced after the cuff deflated. We calculated FMD by the percentage flow-mediated dilation index (FMD%), which is defined as the largest relative change in arterial diameter (20). FMD is affected by CV risk factors and independently predicts the outcome of CV diseases, which is worthy of greater attention in patients with hypothyroidism (21, 22). There were many tools to assess endothelial function including measuring the percent of change in blood flow following heat-mediated vasodilation using laser Doppler flowmetry (10) and peripheral arterial tonometry measuring finger microvascular function. An interesting study compared three noninvasively methods assessing endothelial function and FMD was proven to be a good tool (23).

In the present study, we aimed to determine whether short-term hypothyroidism caused by the thyroxine withdrawal during RAI therapy impaired endothelial function in patients with DTC. Because short-term hypothyroidism was usually accompanied by an abnormal lipid profile (24), we also evaluated the relationships between endothelial function and circulating lipid concentrations.

## Materials and methods

### Patients and RAI procedure

The present study was conducted at the Shanghai Jiao Tong University Affiliated Sixth People’s Hospital and was approved by the local ethics committee. We evaluated patients after they had undergone total thyroidectomy and would accept RAI therapy between March and December 2021 in the Nuclear Medicine Department of Shanghai Sixth People’s Hospital. Patients who satisfied the following criteria were considered for inclusion: they (i) had DTC, confirmed by pathological examination; (ii) underwent total thyroidectomy, and (iii) had never previously undergone RAI therapy. Of the 73 patients who satisfied these criteria, 22 were excluded because of insufficient circulating TSH concentrations (n=2) or missing follow-up data (n=20). Thus, 51 patients who were to undergo RAI therapy were recruited prospectively. All of these had their thyroxine withdrawn for 3–4 weeks prior to RAI to increase their serum TSH concentrations. Their thyroid function and circulating thyroglobulin (Tg) and anti-thyroglobulin antibody (TgAb) concentrations were routinely tested prior to RAI therapy, and neck ultrasonography and chest computed tomography were also performed. An oral dose of ^131^I (30–200 mCi) was empirically administered to the participants, who underwent a ^131^I whole-body scan (^131^I-WBS) 3 days after radioiodine administration. All the participants restarted their TSH suppression therapy 2 days after RAI therapy at the same doses that they had been administering before thyroxine withdrawn.

### Measurements of thyroid function and serum lipid concentrations

Thyroid function and serum lipid concentrations were tested at three time points: the day before thyroxine withdrawal (P_1_), the day before ^131^I administration (P_2_), and 4–6 weeks after RAI therapy (P_3_). The serum concentrations of the following were measured to assess thyroid function: free triiodothyronine (FT_3_), free thyroxine (FT_4_), Tg, TgAb, and TSH. The serum concentrations of triglycerides, cholesterol, low-density lipoprotein (LDL) cholesterol, and high-density lipoprotein (HDL) cholesterol were measured.

### Assessment of endothelial function

Endothelial function was evaluated at three time points. FMD, a non-invasive means of assessing peripheral artery endothelium-dependent dilation using ultrasonography, was used to determine the effect of short-term hypothyroidism on the endothelial function of patients with DTC. FMD was performed according to previously published guidelines (21): the participants fasted for 6 hours, avoided exercise for 24 hours, and refrained from the consumption of caffeine, vitamin C, and alcohol for 12 hours prior to the procedure. If the participants were taking medication for the treatment of underlying diseases, such as hypertension and diabetes, the assessment was performed four half-lives after the preceding dose. The participants relaxed in a quiet, temperature-controlled room for at least 10 minutes before the procedure, to reduce their mental stress level and minimize the effects of any previous physical activity. During the FMD procedure, the blood pressure of each participant was measured using their right arm while in sitting position. To evaluate the brachial artery, a cuff was wrapped around the extended right forearm of each participant while they were in a supine position, an ultrasonographic transducer was applied 3–5 cm above the antecubital fossa, and the baseline diameter of the artery was measured. The cuff was then inflated to a pressure 50 mmHg higher than the participant’s systolic blood pressure (SBP) for 5 minutes, then the recovery of the arterial diameter was monitored on cuff deflation, and the post-ischemic diameter was recorded. The FMD of the brachial artery was calculated as (post-ischemia diameter – baseline diameter)/baseline diameter × 100%. The FMD procedure was performed by a physician with at least 3 years of experience in the use of an Omron UNEXEF38G system, with a probe frequency of 10 MHz. FMD was measured twice for each participant at an interval of 20 minutes, and the mean value was used in further analyses.

### Statistical analysis

Parameters are summarized as mean±standard deviation (SD) or median and interquartile range for continuous data, and as counts or proportions for categorical data. The changes in continuous datasets during therapy were compared using repeated measures analysis of variance (ANOVA) or Friedman’s two-way analysis of variance by rank, as appropriate [general linear model procedure in SPSS]. Prior to performing ANOVA, the assumption of sphericity was tested using Mauchly’s test, and when the sphericity assumption was not met, the multivariate test statistic was determined using Wilks’s lambda test. After Friedman analysis, comparisons between P_1_, P_2_, and P_3_ were performed using repeated Wilcoxon signed rank tests, with the application of the Bonferroni correction to the level of significance for paired groups. Comparisons of categorical data between P_1_, P_2_, and P_3_ were made using the χ^2^ test. Results were considered statistically significant when p<0.05. Data were analyzed using SPSS v.12.0 for Windows (Chicago, IL, USA).

## Results

### Clinical characteristics of the patients

Fifty-one patients were studied, and their clinical characteristics are shown in **Table 1**. The patients had a male-to-female ratio of 1:1.8 and their mean age was 35.3±9.7 years. 50 patients had been diagnosed with papillary thyroid cancer; 39 with classic phenotypes and 11 with variant phenotypes. 1 patient had been diagnosed with follicular thyroid cancer. Their mean body mass index (BMI) was 23.4±3.5 kg/m^2^, and 1 patient had BMI <18 kg/m^2^ and 19 patients had BMI >24 kg/m^2^. 11 patients had CV risk factors (hypertension, smoking, and hyperlipidemia, n=2; diabetes, smoking, and hyperlipidemia, n=1; hypertension and diabetes, n=1; hypertension, n=2; hyperlipidemia, n=5; smoking, n=1). The serum concentration of TSH was >30 mIU/L in all of the patients on the day of ^131^I administration. ^131^I (30–200 mCi) was empirically administered on the basis on the patient’s serum Tg and TgAb concentrations and the results of neck ultrasonography and chest computed tomography. 36 patients were given ^131^I (£100mCi) and the others ^131^I (>100mCi). Post-therapeutic ^131^I-WBS showed that 4 patients had metastases (2 had lymph node metastases and 2 had lung metastases).

**Table 1:** Clinical characteristics of patients.

Patient characteristics	Total sample (N=51)
Age (y) (Mean ± SD)	35.3 ± 9.7
≤55	49(96.1%)
>55	2(3.9%)
Sex (Female)	33(64.7%)
Pathological classification	
PTC(Classic phenotype)	39(76.4%)
PTC(Variant phenotype)	11(21.6%)
FTC	1(2.0)
BMI (kg/m^2^)	23.4 ± 3.5
<18	1(2.0%)
18-24	31(60.8%)
>24	19(37.2%)
Cardiovascular risk factors	
Hypertension	4(7.8%)
Diabetes	2(3.9%)
Hyperlipidemia	8(15.6%)
Smoking	4(7.8%)
None	40(78.4%)
ATA Risk Stratification system (2015)	
Low	12(23.5%)
Intermediate	36(70.6%)
High	3(5.9%)
TNM stage*	
I	49(96.1%)
II	2(3.9%)
RAI dose (mCi)	
≤100	36(70.6%)
>100	15(29.4%)
^131^I-WBS	
Residual thyroid tissue	47(88.3%)
Lymph node metastasis	2(3.9%)
Lung metastasis	2(3.9%)

BMI, body mass index; PTC, papillary thyroid carcinoma; ^131^I-WBS, ^131^I-whole body scan; RAI, radioactive iodine.

* Tumor-node-metastasis (TNM staging is determined by eighth American Joint Cancer Committee TNM staging system).

### Changes in thyroid function, vascular parameters, and lipid concentrations

The thyroid function, vascular, and lipid data at the 3 time points were displayed in **Table 2**. The serum concentrations of TSH and Tg significantly increased between P_1_ and P_2_ and then decreased between P_2_ and P_3_, while the serum concentrations of FT_3_ and FT_4_ showed the opposite trends. These changes imply that the patients experienced a change from subclinical or clinical hyperthyroidism to overt hypothyroidism, but returned to their initial states after the RAI therapy.

**Table 2:** Changes of thyroid function, vascular parameters and lipids.

Characteristics	P1	P2	P3	*p-value*
FMD (%)	8.05 ± 1.55	7.26 ± 1.50*	7.79 ± 1.38	<0.001
Baseline diameter (mm)	2.96 ± 0.57	3.20 ± 0.61*	2.98 ± 0.57	<0.001
SBP (mmHg)	123.1 ± 14.5	120.6 ± 16.1*	117.5 ± 12.7	<0.001
DBP (mmHg)	74.9 ± 9.3	77.3 ± 14.0*	73.2 ± 8.2	0.029
Cholesterol (mmol/L)	4.49[3.79-5.06]	6.22[5.46-7.14]*	4.33[3.79-4.92]	<0.001
Triglyceride (mmol/L)	1.07 [0.75-1.66]	1.71[1.06-2.86]*	1.10[0.81-1.56]	<0.001
HDL-Chol (mmol/L)	1.23 ± 0.34	1.43 ± 0.37*	1.16 ± 0.32	<0.001
LDL-Chol (mmol/L)	2.74 ± 0.76	3.67 ± 1.04*	2.65 ± 0.76	<0.001
FT3 (pmol/L)	4.94 ± 0.94	1.28[1.01-2.00]*	5.27 ± 0.84	<0.001
FT4 (pmol/L)	22.62 ± 4.43	2.261.40-3.21]*	24.60[22.40-28.00]	<0.001
Tg (ng/mL)	0.54[0.04-2.58]	4.20[0.45-30.60]*	0.29[0.04-1.95]	<0.001
TgAb (KIU/L)	14.2[11.6-98.5]	14.3[11.8-124]	14.8[11.7-154.0]	0.783
TSH (mIU/L)	0.32[0.05-1.59]	100.00 [82.90-100.00]*	0.53[0.20-1.65]	<0.001

FMD, flow-mediated dilation; SBP, systolic blood pressure; DBP, diastolic blood pressure; FT_3_, free triiodothyronine; FT_4_, free thyroxine; TSH, thyroid stimulating hormone; HDL, high density lipoprotein; LDL, low density lipoprotein; Tg, thyroglobulin; TgAb, anti-thyroglobulin antibody.

All comparisons made to P_1_:*:p<0.05.

The serum concentrations of lipids (triglyceride, total cholesterol, LDL cholesterol, and HDL cholesterol) were all higher at P_2_ than at P_1_, reflecting thyroxine withdrawal. After the patients restarted their TSH suppression therapy, their serum lipid concentrations decreased, such that they were significantly lower at P_3_ than at P_2_. Vascular parameters, including SBP, diastolic blood pressure, and baseline diameter, showed similar change to the lipids. However, there were no significant differences in thyroid function, vascular parameters, or lipid concentrations between P_1_ and P_3_.

### Changes in endothelial function

**Figure 1:**
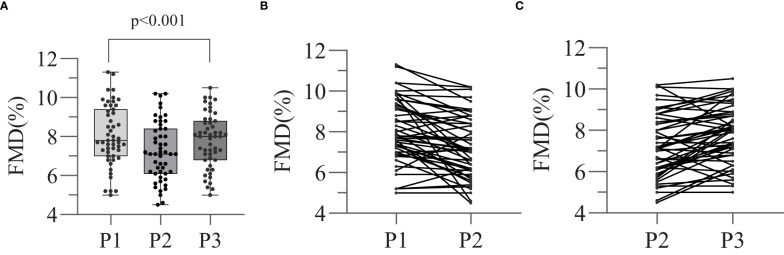
Effect of thyroid dysfunction on endothelial function in different stages. Flow-mediated arterial dilatation (FMD) (endothelin-dependent vasodilatation) was measured at each of the three stages (P_1_, P_2_, P_3_). Different FMD values between three stages were compared by means of repeated measures ANOVA **(A)**. The trends of changing in FMD values of P_1-2_ **(B)** and P_2-3_ **(C)** were also showed separately.

Endothelial function during the various states of thyroid function was assessed by FMD using ultrasonographic imaging, and the results are shown in **Figure 1** and **Table 2**. We found that the FMD of the patients was lower at P_2_ than at P_1_ (7.26±1.50 *vs*. 8.05±1.55, p<0.001), but increased when subclinical or clinical hyperthyroidism developed after the recommencement of TSH suppression therapy, at P_3_ (7.79 ± 1.38 *vs*. 7.26 ± 1.50, p=0.002). There was no significant difference in FMD between P_1_ and P_3_ (8.05 ± 1.55 *vs*. 7.79 ± 1.38, p=0.438). This implies that there was a temporary induction of endothelial dysfunction when the serum TSH concentration increased during the preparation for RAI therapy, which was followed by a gradual recovery when thyroxine therapy was subsequently reinstated.

**Figure 2:**
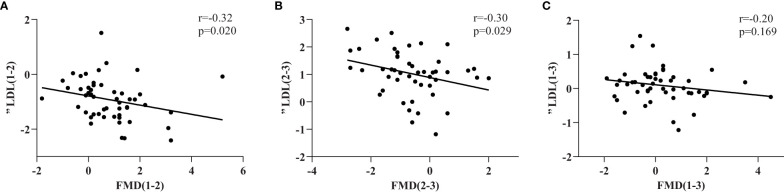
ΔFMD(%) and the correlation with the corresponding change of LDL(ΔLDL) in three stages **(A–C)**.FMD(%) was measured as described in the Methods section and expressed as percentage variation in brachial artery diameter induced by 5 minutes of ischemia.

The relationships between the changes in FMD and serum lipid concentrations during the RAI therapy are shown in **Table 3**. Because of the complexity of the changes in thyroid hormones, we simplified the process into three stages (P_1–2_, P_2–3_ and P_1-3_) and analyzed the relationships separately. We found that the change in FMD (ΔFMD) negatively correlated with the change in LDL cholesterol concentration (ΔLDL) during two stages (P_1–2_, r=−0.326,p=0.020; P_2–3_, r=−0.306, p=0.029) and the correlation plots were shown in **Figure 2**.

**Table 3:** The correlation between the changes of FMD (%) and the corresponding changes of lipids and thyroid hormones.

	ΔFMD_1-2_		ΔFMD_2-3_		ΔFMD_1-3_	
	R	*p-value*	R	*p-value*	R	*p-value*
ΔCholesterol (mmol/L)	-0.22	0.130	-0.31	0.030^*^	-0.18	0.218
ΔTriglyceride (mmol/L)	-0.24	0.083	-0.07	0.640	-0.02	0.899
ΔHDL-Chol (mmol/L)	0.11	0.456	-0.12	0.419	-0.13	0.380
ΔLDL-Chol (mmol/L)	-0.33	0.020*	-0.31	0.029*	-0.20	0.169
ΔFT_3_ (pmol/L)	-0.42	0.770	-0.05	0.709	0.08	0.586
ΔFT4(pmol/L)	-0.12	0.407	0.06	0.703	0.01	0.956
ΔTg (ng/mL)	0.07	0.607	0.10	0.509	-0.08	0.558
ΔTSH (mIU/L)	-0.02	0.910	-0.06	0.695	0.05	0.748

FMD, flow-mediated dilation; TSH, thyroid stimulating hormone; FT3, free triiodothyronine; FT4, free thyroxine; HDL, high density lipoprotein; LDL, low density lipoprotein; Tg, thyroglobulin. *:p<0.05.

12 of the patients showed decreases in FMD of >1% between P_1_ and P_3_. It has been reported that every 1% decrease in FMD was associated with an increase in the incidence of CV events (25, 26). Therefore, we defined a decrease in FMD of >1% as reduced value and placed these 12 participants into a reduced group, and the other 39 into an unreduced group, and compared the clinical characteristics of the two groups. The results were shown in **Table 4**. there were no significant differences in the baseline characteristics in the two groups (age, sex, prevalence of CV risk factors, and BMI).

**Table 4:** The baseline characteristics(P_1_) between Reduced group and Unreduced group.

Baseline characteristics (P_1_)	Reduced group (n=12)	Unreduced group (n=39)	*p-value*
Age (y)	38.42 ± 11.00	35.46 ± 9.26	0.359
Sex (Female)	7(58.3%)	26(66.7%)	0.855
BMI (kg/m^2^)	23.51 ± 3.55	23.41 ± 3.48	0.934
CV risk factors	3(25.0%)	8(20.5%)	0.744
PTC (Classic phenotype)	7(58.3%)	32(82.1%)	0.090
RAI dose (£100mCi)	11(91.7%)	25(64.1%)	0.081
ATA Risk Stratification system (Low/Intermediate/High)	3/9/0	9/27/3	0.616

RAI, radioactive iodine; CV, cardiovascular; *: p<0.05.

## Discussion

In the present study, 51 patients underwent total thyroidectomy for DTC and would accept RAI therapy and experience a short-term hypothyroidism were recruited and most of them were younger than 55 years old except for 2 patients. We have shown that the hypothyroidism which develops following thyroxine withdrawal during RAI therapy caused transient endothelial dysfunction in patients with DTC, and that this was reversed after the reinstatement of TSH suppression therapy. The lipid profile of patients changed alongside these fluctuations in serum thyroid hormone concentrations during the whole process, and the changes in LDL cholesterol negatively correlated with the changes in FMD. In addition, when we compared reduced group with unreduced group, we did not find significant difference in baseline characteristics between the two groups.

DTC accounts for >95% of cases of thyroid cancer, but is associated with a relatively good prognosis because of the availability of effective treatments based on thyroidectomy. TSH suppression, accompanied by thyroid hormone supplementation, is recommended by the American Thyroid Association, which means that patients are in a state of subclinical or clinical hyperthyroidism during the TSH suppression therapy (6). RAI is an effective method of treating thyroid cancer, but requires a sufficient serum TSH concentration, which is usually achieved in the clinic by thyroid hormone withdrawal, such that patients preparing for RAI therapy are hypothyroid during the process of thyroid hormone withdrawal (27). Therefore, these patients experience a change from subclinical or clinical hyperthyroidism to short-term hypothyroidism, and then return to their initial states during the whole RAI therapy process.

Thyroid dysfunction is closely associated with CV disease. It is well known that long-term hypothyroidism is associated with atherosclerosis, with potential mechanisms consisting of hypercholesterolemia, hypertension, and endothelial dysfunction (9). Endothelial dysfunction is an early event in atherosclerosis, and plays an important role in the regulation of hemostasis and thrombosis, local vascular tone, redox balance, and the orchestration of acute and chronic inflammatory reactions within the arterial wall (14). Short-term hypothyroidism were also associated with undesirable cardiovascular effects (28). Endothelial function can be non-invasively assessed using FMD, which is the endothelium-dependent, largely NO-mediated dilatation of arteries in response to induced increases in blood flow and shear stress (29). In the present study, we had characterized the changes in FMD in patients with DTC at the various thyroid function states experienced during the RAI therapy. We found that FMD decreased when overt hypothyroidism developed in patients preparing for RAI therapy and recovered after TSH suppression therapy was restarted. Although differences in this trend existed between the patients, the small sample size prevented definitive conclusions being drawn regarding these.

Erbil et al. recruited 22 patients with non-toxic multinodular goitre treated by total or near-total thyroidectomy and compared the endothelial function of them at different thyroid function states. In this research, endothelial function was impaired caused by the short-term hypothyroidism in the patients and persisted for at least 3 months, and total cholesterol and TSH were independent determinants of it (30). Chang et al. found that the short-term hypothyroidism could cause the clinical, biochemical and cardiovascular changes in DTC patients, but endothelial dysfunction did not present in the patients (31). The different of these studies may be attributed to the individual specificity of the patients with a small sample size and the different research time points, but these researches showed same opinion that short-term hypothyroidism did not cause permanent impairment to patients. Nevertheless, long-term TSH suppression therapy using supraphysiologic thyroid hormone treatment increased heart rate and left ventricular mass led to myocardial strain and impaired diastolic function and reduced arterial elasticity (32). The increased cardiovascular morbidity was confined to patients with a mean TSH level below 0.1mIU/L, but no correlation with the cumulative RAI dose was observed for the risk of all-cause mortality in patients treated with RAI ablation (33). Patients with thyroid cancer usually underwent both long-term supraphysiologic thyroid hormone treatment and transient iatrogenic hypothyroidism. According to our research and the studies above, long-term supraphysiologic thyroid hormone treatment may be more responsible for the developing of cardiovascular issues and needed to be further explored. Then we defined participants who had an FMD at P_3_ that was >1% lower than that at P_1_ as being reduced and the others as being unreduced. The two groups had no significant difference in baseline characteristics, but there was a tendency that patients in reduced group had more invasive PTC subtypes which needed to be further explored. A study with a wide range of ages and larger sample size may be helpful to figure out the difference between two groups.

Previous studies have shown that overt hypothyroidism affects the lipid profiles of patients (34, 35). Consistent with these, we have shown that overt hypothyroidism is associated with changes in serum lipid concentrations (triglyceride, total cholesterol, LDL cholesterol, and HDL cholesterol), but that the serum concentrations of lipids returned to their initial levels after TSH suppression therapy was reinstated. It was worth noting that the change in LDL cholesterol was the only change that correlated with the change in FMD throughout the process of RAI therapy. Although TSH concentration changed during the process, it showed no relationship with the change in FMD, which contrasted with the findings of previous studies, because it was previously reported that the HDL cholesterol concentration is the only independent determinant of FMD (36). Thus, the mechanism that was responsible for the change in FMD during RAI therapy requires further research. The change in LDL cholesterol concentration was found to correlate with the change in FMD across the period of the present study, and therefore the changes in LDL cholesterol that occur during RAI therapy are worthy of further research. In addition, the use of medication might be considered to avoid the sharp rise in LDL cholesterol that occurs in patients during hospitalization to keep the endothelial function. Besides, we only recruited 2 patients older than 55, so whether the endothelial function changes of them differed from young patients were unclear. Vascular endothelial dysfunction is regarded as a primary phenotypic expression of normal human aging. Decreased blood vessel density and endothelial cell subset dynamics appeared during ageing of the endocrine system (37). Another study found the loss of vascular abundance accompanied by the decline in pericytes is a key feature of aging tissues (38). The capacity of expansion of lymphatic vessels is also impaired in aged animals (39). Chronological age steadily impairs endothelial function through reduced endothelial nitric oxide synthase (eNOS) expression/action, accelerated NO degradation and so on (40). The cellular, molecular, and functional changes that occur in the endothelium during ageing can be contributed to the development of cardiovascular diseases (41). So high-quality studies with enough sample size are needed to classify the changes of endothelial function in patients older than 55.

There were several limitations to the present study. Almost all of the patients recruited were younger than 55 from only one medical center making the results applicable more to young patients than old patients. So, multicenter studies with larger sample size from and a wide range of ages should be conducted to verify the appropriateness of our conclusions and extrapolate them to all of the DTC patients. Besides, we only investigated the short-term effects of a single short period of hypothyroidism on endothelial function. However, patients with advanced diseases generally require multiple rounds of RAI therapy, meaning that they can experience several periods of short-term hypothyroidism. It is unknown whether multiple short periods of hypothyroidism can cause permanent damage to vascular endothelial function. We will enlarge the follow-up time and keep tracking the patients to fill this gap in the future work. Furthermore, FMD assesses endothelial-dependent dilatation, whereas nitroglycerin-mediated dilatation (NMD) assesses endothelial-independent dilatation, and in the present study, we did not measure NMD. However, many previous studies have shown that there is no relationship between changes in thyroid function and NMD (16, 17), and nitroglycerin can induce severe hypotension (28). Therefore, we believe that the lack of NMD measurements is not of great importance. Lastly, we only used one tool named FMD to assess the macrovascular endothelial function of the brachial artery of the patients. FMD index did not represent all aspects of endothelial dysfunction and long-term potential effects of the short-term hypothyroidism in patients may exist and needed to be explored.

## Data availability statement

The original contributions presented in the study are included in the article/supplementary material. Further inquiries can be directed to the corresponding authors.

## Ethics statement

The studies involving human participants were reviewed and approved by the Shanghai Jiao Tong University Affiliated Sixth People’s Hospital. Written informed consent to participate in this study was provided by the participants’ legal guardian/next of kin. Written informed consent was obtained from the individual(s), and minor(s)’ legal guardian/next of kin, for the publication of any potentially identifiable images or data included in this article.

## Author contributions

L-YH: Data curation, Formal analysis, Investigation, Writing – original draft. XL, GQ-Z: Data curation, Supervision. CX, C-TS, H-JS: Writing – review and editing.

W-KW, Z-LQ, Q-YL: Resources, review and editing, Supervision, Conceptualization. All authors contributed to the article and approved the submitted version.
